# Genetic Epidemiology of Medication Safety and Efficacy Related Variants in the Central Han Chinese Population With Whole Genome Sequencing

**DOI:** 10.3389/fphar.2021.790832

**Published:** 2022-02-23

**Authors:** Junbo Tian, Jing Zhang, Zengguang Yang, Shuaisheng Feng, Shujuan Li, Shiqi Ren, Jianxiang Shi, Xinyue Hou, Xia Xue, Bei Yang, Hongen Xu, Jiancheng Guo

**Affiliations:** ^1^ BGI College and Henan Institute of Medical and Pharmaceutical Sciences, Zhengzhou University, Zhengzhou, China; ^2^ Precision Medicine Center, Academy of Medical Science, Zhengzhou University, Zhengzhou, China; ^3^ Department of Pharmacy, The Third Affiliated Hospital of Zhengzhou University, Zhengzhou, China; ^4^ School of Information Engineering, Zhengzhou University, Zhengzhou, China; ^5^ The Second Affiliated Hospital of Zhengzhou University, Zhengzhou, China

**Keywords:** pharmacogenomics, genetic polymorphisms, allele frequency, whole-genome sequencing, the central Han Chinese population

## Abstract

Medication safety and efficacy-related pharmacogenomic research play a critical role in precision medicine. This study comprehensively analyzed the pharmacogenomic profiles of the central Han Chinese population in the context of medication safety and efficacy and compared them with other global populations. The ultimate goal is to improve medical treatment guidelines. We performed whole-genome sequencing in 487 Han Chinese individuals and investigated the allele frequencies of pharmacogenetic variants in 1,731 drug response-related genes. We identified 2,139 (81.18%) previously reported variants in our population with annotations in the PharmGKB database. The allele frequencies of these 2,139 clinical-related variants were similar to those in other East Asian populations but different from those in other global populations. We predicted the functional effects of nonsynonymous variants in the 1,731 pharmacogenes and identified 1,281 novel and 4,442 previously reported deleterious variants. Of the 1,281 novel deleterious variants, five are common variants with an allele frequency >5%, and the rest are rare variants with an allele frequency <5%. Of the 4,442 known deleterious variants, the allele frequencies were found to differ from those in other populations, of which 146 are common variants. In addition, we found many variants in non-coding regions, the functions of which require further investigation. This study compiled a large amount of data on pharmacogenomic variants in the central Han Chinese population. At the same time, it provides insight into the role of pharmacogenomic variants in clinical medication safety and efficacy.

## 1 Introduction

Clinical medication efficacy and adverse drug reactions (ADRs) often vary widely among individuals. Pharmacogenomics aims to elucidate the effects of genetic polymorphisms and interindividual differences about the efficacy of medications (Evans and Relling, 1999; Evans and Johnson, 2001). Many studies have demonstrated that gene variants encoding drug-metabolizing enzymes, drug transporters, and drug targets affect drug responses (Choi et al., 2015; Ahmed et al., 2016). The aim of the Pharmacogenomics Knowledge base (PharmGKB; https://www.pharmgkb.org) is to collect and analyze data and then disseminate knowledge on the impact of genetic variations associated with drug responses. PharmGKB provides clinical information on genotype-phenotype relationships and variant–drug associations based on well-defined criteria and careful literature reviews.

Traditional methods to detect drug reaction-related genetic polymorphisms include PCR and microarray-based techniques (Hodel et al., 2009; Burmester et al., 2010). Although these methods are cost-effective and easy to implement, they focus on the most common pharmacogenomic variants rather than identifying novel or rare polymorphisms associated with individual differences in drug responses. Next-generation sequencing (NGS) technology addresses the shortcomings of conventional detection methods. Whole-exome sequencing (WES) and whole-genome sequencing (WGS) can be used not only for the diagnosis of Mendelian diseases but also for the comprehensive investigation of drug response-related variants in individuals (Katsila and Patrinos, 2015; Ji et al., 2018). Given the decreasing cost of NGS, many studies have applied WES and WGS to pharmacogenomic research and obtained novel insights (Altman et al., 2013; Ahn and Park, 2017; Sivadas et al., 2017; Sivadas and Scaria, 2018; Choi et al., 2019; Caspar et al., 2020).

Pharmacogenomically relevant variants, in terms of drug efficacy and adverse effects, vary widely in frequency among global populations (Yasuda et al., 2008; Ramos et al., 2014). Moreover, some drugs with safe and effective doses for ethnicities with certain genetic variants are not appropriate for others (Rieder et al., 2005; Lam et al., 2016). Therefore, it is essential to widen the scope of pharmacogenomic research to encompass populations worldwide and increase the evidence base for precision medicine.

China comprises multiple ethnicities. For safe, reasonable, and precise personalized therapy, comprehensive pharmacogenetic analysis of the Chinese population is required. However, most studies have focused only on the frequencies of common variants in several essential genes in the Chinese population (Qian et al., 2013; Hu et al., 2017; Liu et al., 2019; Qi et al., 2020a). For example, Dai et al. and Hu et al. systematically investigated polymorphisms in the cytochrome P450 (CYP) genes *CYP2C9* and *CYP2C19*, respectively, in the Han Chinese population (Hu et al., 2012; Dai et al., 2014). Although the sample sizes were large, both of those studies were concerned with only one gene, and variants in intronic regions were not revealed due to the methods’ limitations. Qi et al. (2020b) assessed the genetic variations in 57 CYP and cytochrome P450 oxidoreductase genes in a large-scale WGS study based on the Chinese Millionome database; however, the shallow sequencing depth may have led to rare variants being missed.

This study investigated the distribution of pharmacogenomic variants in the central Han Chinese population using high-depth WGS and compared the allele frequencies with those in other global populations. We also comprehensively analyzed the allele frequencies of variants with PharmGKB annotations. To the best of our knowledge, this is the first comprehensive pharmacogenomic study conducted in a Chinese population.

## Materials and Methods

### Study Population

This study enrolled 487 healthy subjects (198 males and 289 females) aged 18–60 years. The subjects were not biologically related and were all Han Chinese. Based on their medical records, all of the participants were healthy. Furthermore, they all signed informed consent forms before any blood samples were collected. The ethics committee of Zhengzhou University approved the study protocol (reference number: ZZURIB 2019-002).

### Whole-Genome Sequencing

Peripheral venous blood samples (3–4 ml) were collected into EDTA anticoagulant tubes. Genomic DNA was extracted from white blood cells using the GenMagBio Genomic DNA Purification kit (GenMagBio, Changzhou, China). The concentration and purity of the genomic DNA were measured using the NanoDrop One instrument (Thermo Fisher Scientific, Waltham, MA, United States), and the quality of the DNA was determined by 1% agarose gel electrophoresis.

Genomic DNA was fragmented (∼400 bp) using sonication. The fragmented DNA was then end-repaired, ligated to adapters, and PCR-enriched using the VAHTS Universal DNA Library Prep Kit (Vazyme Biotech Co. Ltd., Nanjing, China) according to the manufacturer’s protocol. The resulting DNA libraries were sequenced using the HiSeq 4000 platform (Illumina Inc., San Diego, CA, United States) operating in paired-end 150 bp mode (∼30×) at the Precision Medicine Center of Zhengzhou University (Zhengzhou, China).

### Bioinformatics Analysis

Sequencing adapters and low-quality reads were trimmed from raw reads using Trimmomatic (Bolger et al., 2014). Clean reads were aligned to the human reference genome hg19 using BWA-MEM (version 0.7.17-r1188) (Li, 2013). Single nucleotide variants and minor insertion/deletions were characterized using the Genome Analysis Toolkit (version 4; GATK4) HaplotypeCaller (DePristo et al., 2011). Variant annotation was performed using SnpEff and Vcfanno and several annotation databases (Cingolani et al., 2012; Liu et al., 2016; Pedersen et al., 2016). All of the bioinformatics analysis steps were performed within the framework of bcbio-nextgen (https://github.com/bcbio/bcbio-nextgen).

### Pharmacogenomic Variant Analysis Workflow

#### Variants in Pharmacogenes

**FIGURE 1:**
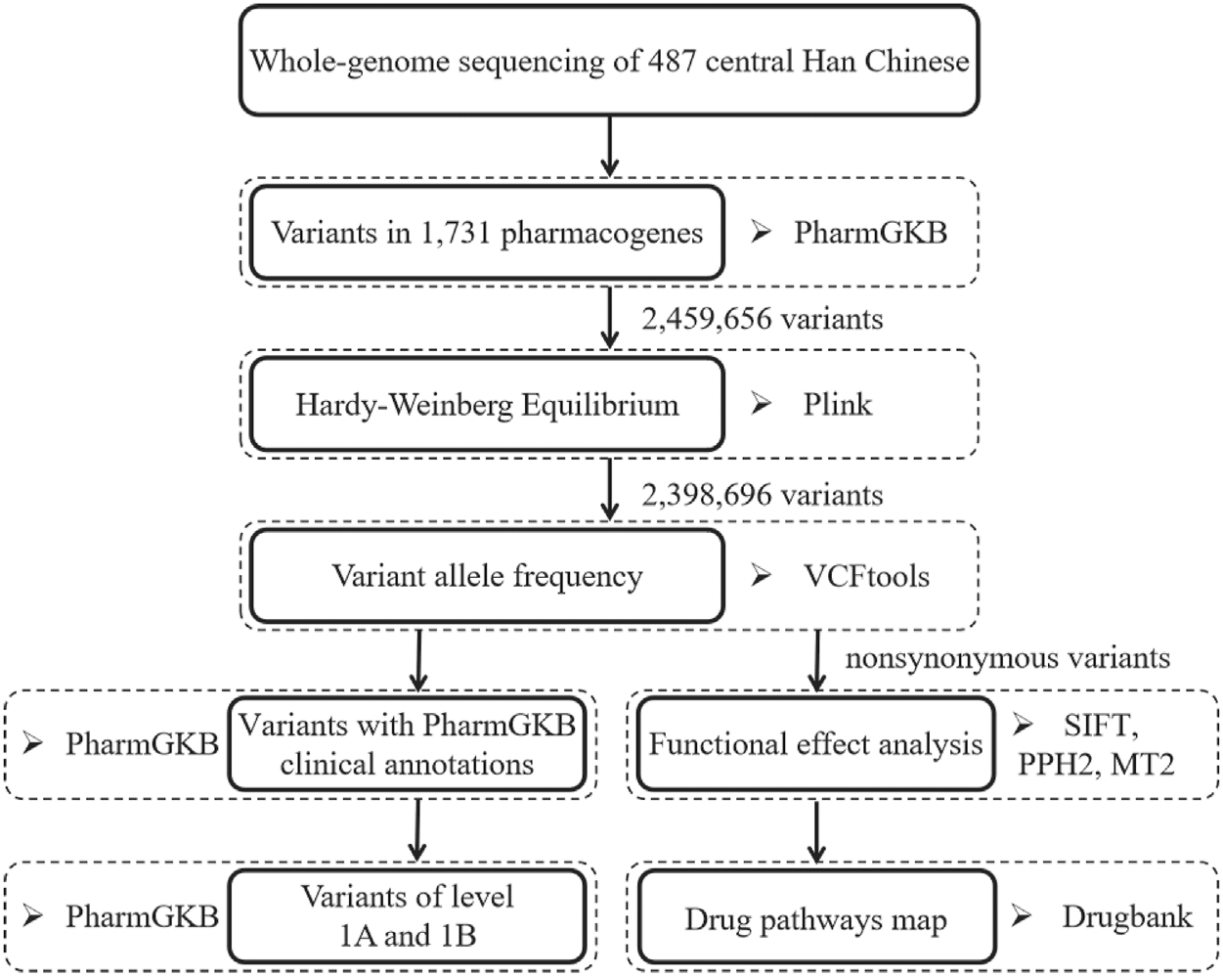
The overview of analysis workflow for pharmacogenomic variant in the central Han Chinese population. PPH2, PolyPhen2. MT2, Mutation Taster 2.

We downloaded the gene list from the PharmGKB database and identified 1,731 PharmGKB-annotated genes using the “Has Variant Annotation” search field (Whirl-Carrillo et al., 2012). The chromosomal locations of the pharmacogenes were obtained from the NCBI database (https://www.ncbi.nlm.nih.gov/). We extracted all variants (*n* = 2,459,656) in the 1,731 pharmacogenes from 487 WGS datasets. Variants with annotation information in the Single Nucleotide Polymorphism database (dbSNP; version 151) were defined as known variants, while those without dbSNP accession IDs were considered novel variants (Sherry et al., 2001). The analysis workflow is summarized in Figure 1.

#### Hardy–Weinberg Equilibrium and Variant Allele Frequency Calculation

We assessed Hardy–Weinberg equilibrium (HWE; *p* < 0.05 with false discovery rate [FDR] adjustment) using PLINK v1.9 (Chang et al., 2015) and obtained 2,398,696 (97.52%) variants for downstream analysis. We calculated the variant allele frequencies (VAF) of the 2,398,696 variants in the central Han Chinese population using VCFtools (Danecek et al., 2011).

#### Prediction of Potentially Deleterious Variants

Nonsynonymous variants (missense variant, start loss, stop loss, and stop gain) were examined for deleterious effects on the encoded proteins using SIFT (Ng and Henikoff, 2003), PolyPhen2 (Adzhubei et al., 2010), and MutationTaster2 (Schwarz et al., 2014). Variants were classified as potentially deleterious based on the predictions of at least two tools (i.e., as “damaging” by SIFT, “probably damaging” by Polyphen2, and “disease-causing” by MutationTaster2).

#### Construction and Visualization of a “Drug Pathway Map”

Pharmacogenes with a deleterious variant and allele frequency >10% in our population were mapped to drugs in the DrugBank (Wishart et al., 2018). Then, a Sankey flow diagram was constructed using Microsoft Power BI (Microsoft Corp., Redmond, WA, United States).

#### Variants With PharmGKB Clinical Annotations

We downloaded the clinical annotations for pharmacogenomic variants from PharmGKB. The distributions of the 2,635 unique single nucleotide polymorphisms (SNPs) in our study population were analyzed (Whirl-Carrillo et al., 2012). The allele frequencies of variants considered to have a higher level of clinical evidence (levels 1A and 1B) (Whirl-Carrillo et al., 2012) were compared with those in other populations included in the 1000 Genomes Project phase 3 (1KG3) (ftp://ftp.ncbi.nlm.nih.gov/1000genomes/ftp/phase3/data/) (Genomes Project et al., 2015) and genome Aggregation database (gnomAD) (https://gnomad.broadinstitute.org/) (Karczewski et al., 2020) by chi-square test.

#### Comparison of Allele Frequencies with Those in Populations from 1KG3 and gnomAD

The variant frequencies for the central Han Chinese population were extracted based on an HWE test of the level 1A and 1B variants in PharmGKB. The frequency information in our population was compared with all populations as a whole and the East Asian populations in the gnomAD and 1KG3 database, which is illustrated by a scatterplot. The variant frequencies of high evidence levels (1A or 1B) are illustrated as a bubble diagram. The scatterplots and bubble diagrams were generated by the R package ggplot2 (R version 4.0.2) (Wickham et al., 2016). Among potentially deleterious variants, common variants (VAF> 10%) compared with other populations were visualized as a heatmap. The heatmaps were produced using the R package ComplexHeatmap (R version 4.0.2) (Gu et al., 2016).

## Results

### Summary of the Variant Analysis

This study analyzed a WGS dataset comprising 487 central Han Chinese individuals. Specifically, we focused on the variants in 1,731 drug response-related genes. Quality control (QC) is essential for raw NGS data. In this study, our sample’s average, minimum, and maximum Q30 values were 97.19, 95.00, and 98.31%, respectively. Sequencing reads were mapped to the human reference genome (GRCh37); the average sequencing depth data are summarized in Table 1. Coverage refers to the proportion of the genome that has been sequenced (Table 1).

**TABLE 1:** Summary of Quality control (QC).

	Q30 (%)	Map (%)	Depth (%)	Coverage (%)	
				1×	10×
Average	97.19	99.85	28	99.11	96.72
Minimum	95.00	99.09	22	98.00	94.00
Maximum	98.31	99.90	63	100.00	100.00

After the HWE tests, a total of 2,398,696 variants in 1,731 pharmacogenes were obtained, of which 80.11% were known (i.e., had rs IDs in dbSNP v151), and 476,984 variants were novel. Variant annotation revealed 18,907 missense variants, 13,923 synonymous variants and 1,746,470 intronic variants (72.81%). The variant annotations are summarized in Supplementary Table S1.

Allele frequency analysis of the 2,398,696 variants in the central Han Chinese population showed that a large number of variants were rare (65.23%; VAF <1%), 231,447 were low frequency (9.65%, VAF = 1–5%), and 602,586 were common (25.12%; VAF >5%) (Supplementary Figure S1).

### Potentially Deleterious Variants in Pharmacogenes Among the Central Han Chinese Population

To achieve a comprehensive understanding of the 1,731 drug response-related pharmacogenetic variants identified in our central Han Chinese population, we used SIFT, PolyPhen-2, and MutationTaster2 to predict the functional impact of 19,368 nonsynonymous variants. A total of 5,723 variants were predicted to be potentially deleterious using at least two of the tools (Table 2, Supplementary Figure S2); these 5,723 variants, 1,281 of which are novel, may impair the function of 1,316 genes (Supplementary Table S2). Of the 5,723 variants, 149 were classified as common (VAF >5%) and 5,253 as rare (VAF <1%); 4,023 of the rare variants were found in only one person. The allele frequencies of 47 of the 1,281 novel variants were >1%; the others were classified as rare (Supplementary Figure S3).

**TABLE 2:** Summary of functional effect prediction of nonsynonymous variants.

Tool	Predicted effect	No. of SNVs	Genes
SIFT	Damaging	7,373	1,399
PolyPhen-2	Probably damaging	3,622	1,098
MutationTaster2	Disease-causing	8,777	1,467
Total potential deleterious SNVS (prediction by at least 2 out of 3 tools)		5,723	1,316

**FIGURE 2:**
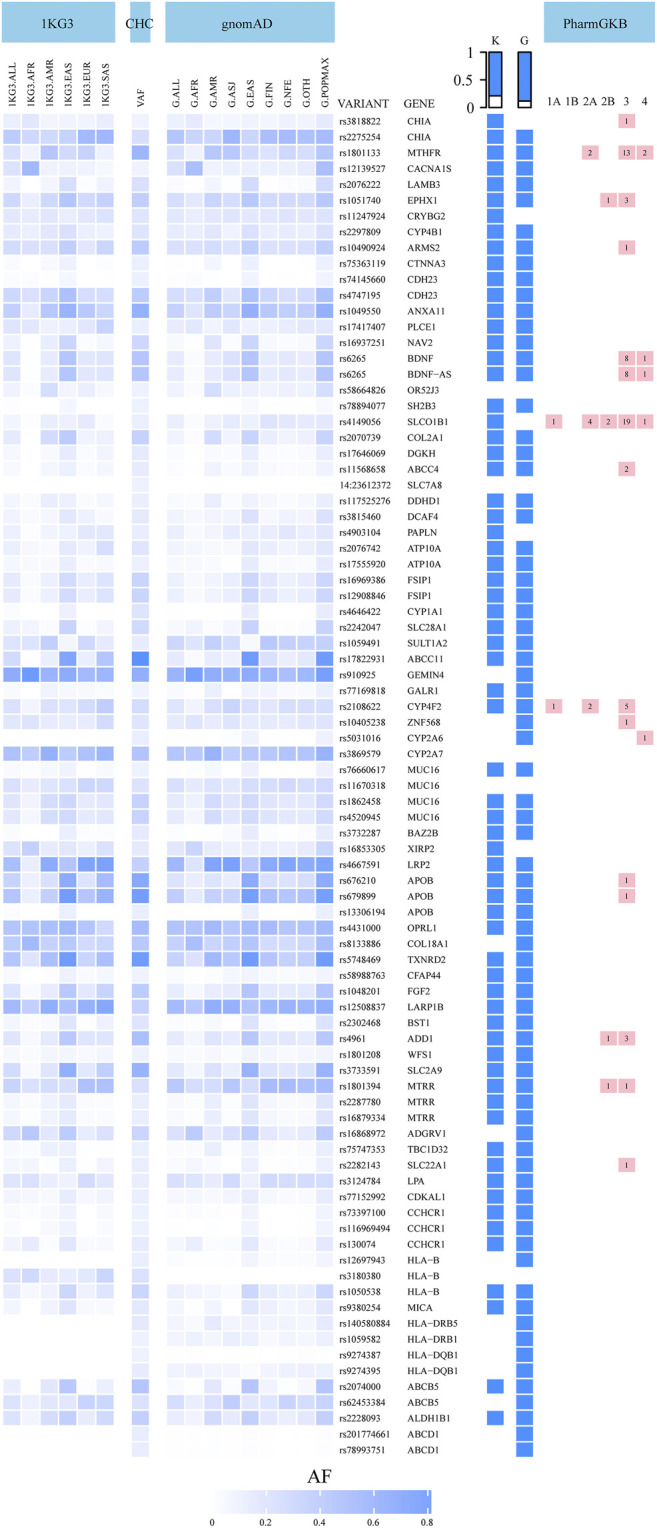
Allele frequencies of 85 most common potentially deleterious variants in the central Han Chinese population compared to global populations in 1000 Genomes Project phase 3 (1KG3) and gnomAD databases (G). The table on the right of the heatmap: VARIANT: variant name, GENE: gene name, K: blue color indicates that the allele frequency in our study is different from the 1KG3.ALL frequency (q < 0.05), G: blue color indicates that the allele frequency in our study is different from the G.ALL frequency (q < 0.05), the last column represents the number of clinical annotations at various levels of evidence of 16 variants in the PharmGKB. CHC: central Han Chinese population; VAF: variant allele frequency.

Among the 5,723 potentially deleterious variants, the 85 classified as common (VAF >10%) affect the function of 67 genes. We present the allele frequencies of these 85 variants in our central Han Chinese population, along with those in the other populations included in the 1KG3 and gnomAD databases, in Figure 2 and Supplementary Table S2. Comparison of the allele frequencies revealed that 67 and 75 variants differed significantly between our dataset and the 1KG3 and gnomAD database populations, respectively (FDR-adjusted p-values < 0.05). For example, variant rs4646422, which impairs the function of *CYP1A1*, was highly prevalent (VAF = 0.2228) in our central Han Chinese population compared with the other populations (1KG3.ALL, VAF = 0.0242; G.ALL, VAF = 0.0077; 1KG3.EAS, VAF = 0.1151; G.EAS, VAF = 0.1535). The *SH2B3* gene has a deleterious variant, rs78894077, with high frequency among East Asian populations (our cohort, VAF = 0.1140; 1KG3.EAS, VAF = 0.0635; G.EAS, VAF = 0.0546); however, it is largely absent from other populations.

In total, 16 of the 85 potentially deleterious variants have clinical annotations in the PharmGKB database (Whirl-Carrillo et al., 2012). These 16 genes include 5 “very important pharmacogenes,” *CYP2A6*, *CYP4F2*, *MTHFR*, *SLC22A1*, and *SLCO1B1*, which are involved in the metabolism and transport of many pharmacological agents. The 16 variants were associated with 80 clinical annotations with varying levels of evidence. The allele frequencies of 14 of these 16 variants were significantly different from those in the global populations included in the 1KG3 and gnomAD databases (Figure 2, Supplementary Table S2). The rs1801133 variant in the *MTHFR* gene was associated with 17 clinical annotations. *MTHFR*, which affects the efficacy and toxicity of antineoplastic drugs such as methotrexate, carboplatin, and cisplatin (level 2A), was more prevalent among our central Han Chinese compared with the other global and East Asian populations (our cohort, VAF = 0.6273, 1KG3.ALL, VAF = 0.2454; G.ALL, VAF = 0.2573; 1KG3.EAS, VAF = 0.2956; G.EAS, VAF = 0.2884). The *MTRR* variant rs1801394 is involved in the toxicity of, and ADRs to, methotrexate (level 2B); its prevalence in our population was lower than that in the global populations in the databases and similar to that in the majority of the other East Asian populations (our cohort, VAF = 0.2536; 1KG3.ALL, VAF = 0.3642; G.ALL, VAF = 0.4622; 1KG3.EAS, VAF = 0.2629; G.EAS, VAF = 0.2805). The 69 variants without clinical annotations involved 55 genes, including the “very important pharmacogenes” *HLA-B* and *CACNA1S*. Allelic variants in *HLA-B* have been associated with ADRs to abacavir and carbamazepine, among other drugs.

### Drug Pathway Analysis of Disrupted Pharmacogenes in the Central Han Chinese Population

**FIGURE 3:**
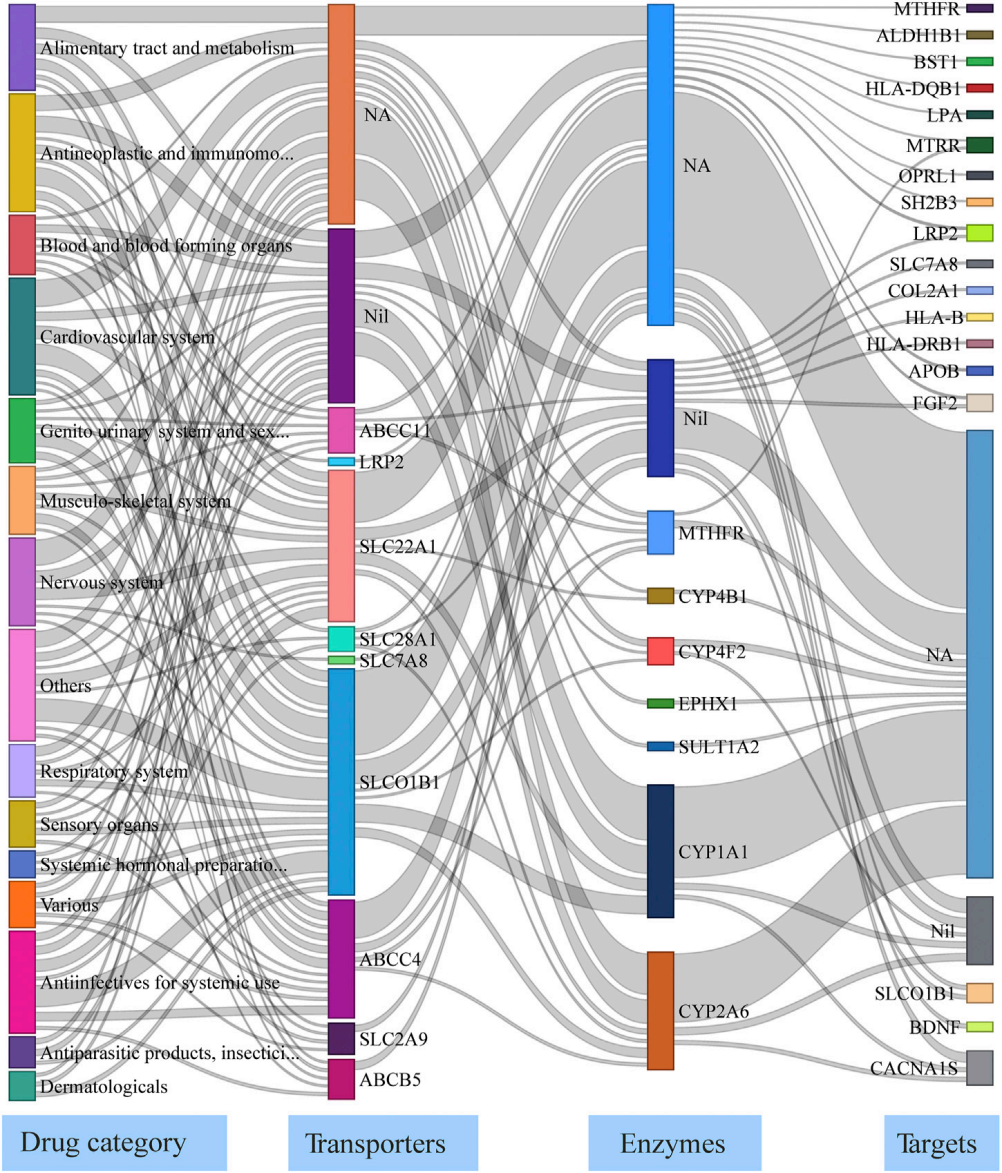
Drug pathway map describing functionally-impaired pharmacogenes in the central Han Chinese population. The four columns in the map represent the major drug category affected by putatively deleterious variants, transporter/carrier genes, enzyme genes, and target genes. NA: None Affected; Nil: No known genes.

To analyze the effect of disrupted pharmacogenes in drug pathways, we mapped the 67 genes with deleterious variants and a VAF >10% to drugs in the DrugBank database. In total, 416 drugs were associated with 32 genes; the drug pathways are presented as a Sankey flow diagram (Figure 3, Supplementary Table S3). These 32 genes harbored the 40 most common deleterious variants and were associated with two carriers, 10 transporter, seven enzyme, and 20 target genes. The drug pathway map includes a wide range of drug classes (e.g., cardiovascular, antineoplastic, and immunomodulating agents). As an example, bezafibrate, a hypolipidemic agent, may be affected by transport and metabolic functions because of its transporter gene (*SLCO1B1*) and primary metabolizing enzyme (*CYP1A1*) both contained deleterious variants in more than 10% of our central Han Chinese population. Nifedipine is a dihydropyridine L-type calcium channel blocker used to treat hypertension; its target gene (*CACNA1S*) and primary enzyme genes (*CYP1A1* and *CYP2A6*) all had deleterious variants in our population. These findings shed light on the interplay between drug-related genes in drug pathways and drug responses in central Han Chinese populations.

### Overall Distribution of Variants With PharmGKB Clinical Annotations

**FIGURE 4:**
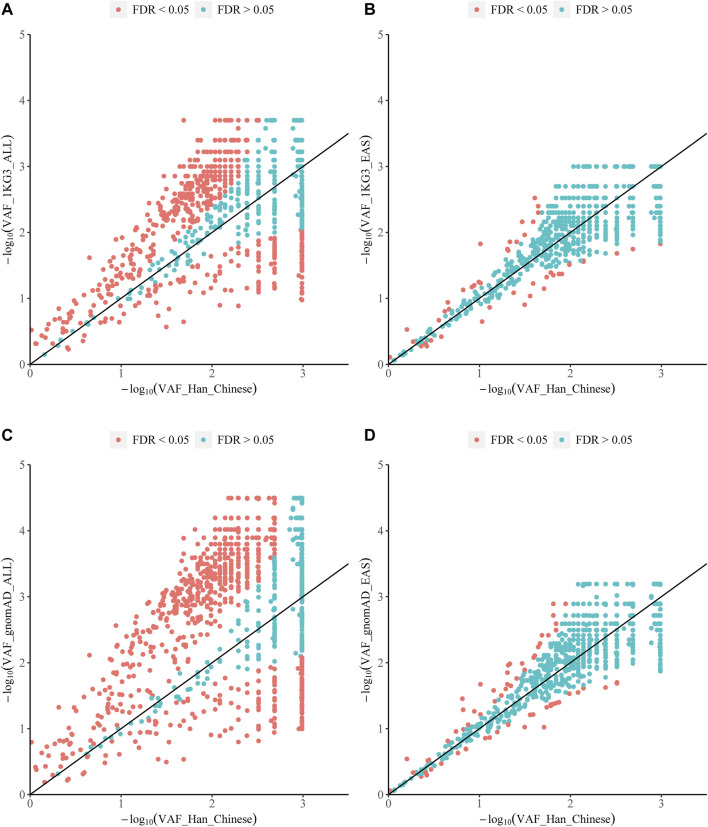
Compared with the mutation frequencies of all populations in the 1KG3 and gnomAD databases, 1,790 and 1,920 genetic mutations are statistically different (FDR <0.05). Compared with the mutation frequencies of the East Asian populations in the 1KG3 and gnomAD databases, respectively. There were statistical differences between 393 and 333 genetic variants (FDR <0.05). VAF_Han_Chinese: variant allele frequency in the central Han Chinese population; VAF_1KG3_ALL: Variant Allele Frequency for all populations in 1KG3; VAF_1KG3_EAS: Variant Allele Frequency for East Asian population in 1KG3; VAF_gnomAD_ALL: Variant Allele Frequency for all populations in gnomAD; VAF_gnomAD_EAS: Variant Allele Frequency for East Asian population in gnomAD.

To investigate the overall distribution of variants with PharmGKB clinical annotations among our central Han Chinese population, we attempted to match the 2,635 SNP variants from PharmGKB to the list of 2,398,696 variants identified in our cohort; 2,139 (81.18%) clinically relevant variants were matched (Supplementary Table S4). Among these 2,139 variants, the allele frequencies of 85.83% (N = 1,836) were >5%, while 7.01% (*n* = 150) were rare in our population (Supplementary Figure S4). Compared with all populations in the 1KG3 and gnomAD databases, the frequencies of 1,790 and 1,920 variants, respectively, were significantly different (FDR <0.05). The frequencies of 393 and 333 variants were also significantly different from those in the East Asian populations in the 1KG3 and gnomAD databases, respectively (FDR <0.05) (Figure 4). Of the 2,139 variants, 24 (30 clinical annotations) had high evidence levels (1A or 1B) and were related to 15 genes and 34 therapeutic agents (Table 3, Supplementary Table S4).

**TABLE 3:** Clinical annotations of 30 variants with a higher level of evidence (Level 1A and 1B) in PharmGKB.

Variant	Gene	Type	Level of evidence	Chemicals	VAF (%)
rs1057910	*CYP2C9*	Dosage	1A	Warfarin	4.11
rs1057910	*CYP2C9*	Metabolism/PK	1A	Celecoxib	4.11
rs1057910	*CYP2C9*	Toxicity/ADR	1A	Phenytoin	4.11
rs115545701	*CFTR*	Efficacy	1A	Ivacaftor	0.10
rs116855232	*NUDT15*	Dosage, Toxicity/ADR	1A	Azathioprine, mercaptopurine	13.46
rs12248560	*CYP2C19*	Dosage, Efficacy,Toxicity/ADR	1A	Clopidogrel	0.62
rs12777823		Dosage	1A	Warfarin	30.49
rs12979860	*IFNL3, IFNL4*	Efficacy	1A	Peginterferon alfa-2a/2b, ribavirin, telaprevir	6.16
rs12979860	*IFNL3, IFNL4*	Efficacy	1A	Peginterferon alfa-2a/2b,ribavirin	6.16
rs1799853	*CYP2C9*	Dosage	1A	Warfarin	0.31
rs2108622	*CYP4F2*	Dosage	1A	Warfarin	27.93
rs2228001	*XPC*	Toxicity/ADR	1B	Cisplatin	65.09
rs267606617	*MT-RNR1*	Toxicity/ADR	1B	Amikacin	0.21
				Aminoglycoside antibacterials	
				Gentamicin	
				Kanamycin	
				Neomycin	
				Streptomycin	
				Tobramycin	
rs28399504	*CYP2C19*	Efficacy	1A	Clopidogrel	0.21
rs3745274	*CYP2B6*	Dosage	1A	Efavirenz	18.38
rs3892097	*CYP2D6*	Dosage, Toxicity/ADR	1A	Amitriptyline	0.62
				Antidepressants	
				Clomipramine	
				Desipramine	
				Doxepin	
				Imipramine	
				Nortriptyline	
				Trimipramine	
rs4149056	*SLCO1B1*	Toxicity/ADR	1A	Simvastatin	12.92
rs4244285	*CYP2C19*	Efficacy, Toxicity/ADR	1A	Clopidogrel	28.94
rs4244285	*CYP2C19*	Efficacy	1A	Amitriptyline	28.94
rs4986893	*CYP2C19*	Efficacy, Toxicity/ADR	1A	Clopidogrel	5.03
rs7294	*VKORC1*	Dosage	1B	Warfarin	6.98
rs75541969	*CFTR*	Efficacy	1A	Ivacaftor	0.10
rs776746	*CYP3A5*	Dosage, Metabolism/PK	1A	Tacrolimus	26.80
rs7900194	*CYP2C9*	Dosage, Toxicity/ADR	1A	Warfarin	0.10
rs8099917	*IFNL3*	Efficacy	1B	Peginterferon alfa-2a/2b,ribavirin, telaprevir	5.03
rs8099917	*IFNL3*	Efficacy	1B	Interferons, peginterferon alfa-2a/2b,ribavirin	5.03
rs887829	*UGT1A1*	Other	1A	Atazanavir	11.60
rs9923231	*VKORC1*	Dosage	1A	warfarin	92.71
rs9923231	*VKORC1*	Dosage	1B	Acenocoumarol, phenprocoumon	92.71
rs9934438	*VKORC1*	Dosage	1B	Warfarin	92.71

**FIGURE 5:**
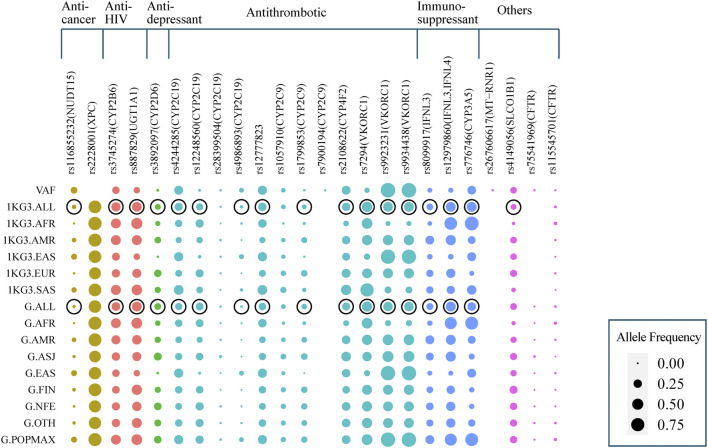
Comparison of the Han Chinese allele frequencies of clinically significant PharmGKB variants with populations included in 1000 Genomes Project phase 3 (1KG3) and gnomAD databases (G). The variants are arranged according to the category of drugs they affect. The size of the solid circle represents the allele frequencies ranging from 0.00 to 1.00. The black outer ellipse represents that the variant allele frequency of Han Chinese has a statistical difference compared to global population averages (1KG3.ALL and G.ALL).

We then compared the allele frequencies of 24 clinically significant variants in our study population with those in the global populations included in the 1KG3 and gnomAD databases (Figure 5). The frequencies of 17 alleles were significantly different from those of the average global population (FDR-adjusted p-value < 0.05). However, only five variants in our population showed significant differences compared with the other East Asian populations. The *VKORC1* variant rs7294, associated with warfarin dosage, showed a lower frequency among our central Han Chinese population (VAF = 0.0698) compared with the global populations (1KG3.ALL, VAF = 0.4197; G.ALL, VAF = 0.3948) and other East Asian populations (1KG3.EAS, VAF = 0.1121, G.EAS, VAF = 0.1013). In addition, the other variants in *VKORC1*, rs9923231 and rs9934438, showed significantly higher prevalences (VAF = 0.9271 and 0.9271, respectively) in our population compared with the global populations (1KG3.ALL, VAF = 0.3556 and 0.3558, respectively; G.ALL, VAF = 0.3260 and 0.3261, respectively). The *NUDT15* variant rs116855232, associated with azathioprine and mercaptopurine dosage, toxicity, and ADRs, was more widely observed among the central Han Chinese population (VAF = 0.1346) compared with the global populations (1KG3.ALL, VAF = 0.0395; G.ALL, VAF = 0.0110) and other East Asian populations (1KG3.EAS, VAF = 0.0952; G.EAS, VAF = 0.0972).

## Discussion

The allele frequencies of pharmacogenomic markers of drug efficacy and toxicity vary among ethnicities (Ramos et al., 2014). Genetic variants can impact medication doses and therapeutic decision-making, for which there is a need to avoid ADRs (Limdi et al., 2008; Li et al., 2018). However, many studies only focused on a few variants in several commonly investigated genes; thus, rare variants may have been missed, resulting in inappropriate drug prescriptions in some cases. Therefore, it is necessary to expand the scope of pharmacogenomic research to encompass multiple ethnic populations. WES and WGS provide an opportunity for a more comprehensive analysis of pharmacogenomic profiles (Petersen et al., 2017). In the present study of 487 central Han Chinese individuals, we used high-depth WGS data to assess the allele frequencies of variants with PharmGKB clinical annotations, with deleterious variants potentially affecting the function of pharmacogenes.

The screening of variants with PharmGKB clinical annotations is of high clinical utility; 2,139 (81.18%) clinically relevant variants were found in our population. Among the 119 variants in PharmGKB with a higher level of evidence (1A or 1B), only 24 SNPs were found in our central Han Chinese population, whereas a large proportion of the variants (79.83%) were not detected. The differences among the populations demonstrated the genetic heterogeneity among ethnic groups. The 24 variants with clinical annotations involved 14 genes, such as the CYP gene family, *VKORC1*, etc. According to research by Biswas (2021), the phenotype of the *CYP2C19* gene is divided into extensive metabolizers (EM), poor metabolizers (PM), intermediate metabolizers (IM), and ultrarapid metabolizers (UM). Among them, UM (CYP2C19*1/*17; CYP2C19* 17/*17) and PM (CYP2C19*2/*2; CYP2C19*3/*3, CYP2C19*2/*3) were considered high risk phenotypes. UM were prevalent high in Africa (33.7%) and low in the Central Han Chinese Population (1.2%). The prevalence of PM in South Asia and the Central Han Chinese Population is similar, about 11%. Further research is needed to fully understand the polymorphisms in the Han Chinese population.”

Functional predictions of the variants in 1,731 pharmacogenes revealed that the functions of 1,316 genes may be affected by 5,723 potentially deleterious variants, 5253 (91.77%) of which were classified as rare. This shows the importance of NGS for discovering rare variants that may account for a large proportion of the unexplained interindividual differences in metabolic phenotypes observed for some drugs (Ingelman-Sundberg et al., 2018). Among the 5,723 deleterious variants in this study, 1,281 novel variants were identified; their effects on the functions of pharmacogenes need to be elucidated in further studies. Finally, we highlighted the differences in the allele frequencies of 85 common (VAF >10%) deleterious variants between our cohort and other global populations. This information could facilitate optimal drug selection and dosing regimens.

In conclusion, this is the first study to analyze pharmacogenomic variants in the central Han Chinese population comprehensively. In total, 2,139 clinically relevant variants were identified, of which 24 had high levels of evidence (1A or 1B). We also found that 5,723 of 2,398,696 variants are potentially deleterious, of which 1,281 are novel. We compared the allele frequencies of 85 common (VAF >10%) deleterious variants with those in other populations. The differences in allele frequencies among the populations demonstrated the genetic heterogeneity among ethnic groups. WGS shows great potential based on the results of our study but also faces challenges such as difficulty in interpreting variants of unknown significance in drug-related genes. A comprehensive understanding of genetic polymorphisms at the population level is essential for safe, rational, and effective utilization of drugs and for precision medicine. However, the effects of certain novel and rare pharmacogenetic variants need to be verified by functional experiments and clinical studies.

## Data Availability

The raw sequencing data supporting this article cannot be placed in public repository due to national legislation/guidelines, specifically the Regulation of the People's Republic of China on the Administration of Human Genetic Resources (http://www.gov.cn/zhengce/content/2019-06/10/content_5398829.htm, http://english.www.gov.cn/policies/latest_releases/2019/06/10/content_281476708945462.htm). As required by the funding bodies, the raw sequencing data were deposited in the National Supercomputing Center in Zhengzhou. Please email nscc@zzu.edu.cn for detailed application guidance. The accession code can be obtained by emailing the corresponding authors upon reasonable request.

## Abbreviations

ADR, adverse drug reactions; gnomAD, genome Aggregation Database; NGS, next-generation sequencing; SNVs, single nucleotide variants; VAF, variant allele frequency in the central han chinese population; VIPs, very important pharmacogenes; WES, whole-exome sequencing; WGS, whole-genome sequencing; 1KG3, 1000 genomes project phase 3.
