# Supplementary material for: Genetic Epidemiology of Medication Safety and Efficacy Related Variants in the Central Han Chinese Population With Whole Genome Sequencing
Source: Front Pharmacol. 2022 Feb 23;12:790832. doi: 10.3389/fphar.2021.790832 (PMC8906509; doi:10.3389/fphar.2021.790832)
Supplement: Supplementary file 3 [file Table1.docx]

# SUPPLEMENTARY TABLES

## Supplementary Table 1: Variant annotation statistics for 2,398,696 variants in 1,731 pharmacogenes analyzed using SnpEff.

| **Summary** | **Count** |
| --- | --- |
| Total variants | 2,398,696 |
| Known variants(dbSNP151) | 1,921,712 |
| Novel variants | 476,984 |
|  | |
| **Type of variants** | **Total SNVs (Novel)** |
| missense variant | 18,907(3,644) |
| Stop gained | 353(92) |
| Stop lost | 48(15) |
| Start lost | 60(24) |
| Synonymous variant | 13,923(2,973) |
| 3_prime_UTR_variant | 35,533(8,084) |
| 5_prime_UTR_variant | 9,938(2,320) |
| Intergenic variant | 16,001(3,247) |
| Intron variant | 1,746,470(343,491) |
| Splice variant | 4,902(973) |
| Downstream gene variant | 207,744(41,234) |
| Upstream gene variant | 285,224(58,879) |
| others | 59,593(12,008) |
