# Supplementary material for: Genetic Epidemiology of Medication Safety and Efficacy Related Variants in the Central Han Chinese Population With Whole Genome Sequencing
Source: Front Pharmacol. 2022 Feb 23;12:790832. doi: 10.3389/fphar.2021.790832 (PMC8906509; doi:10.3389/fphar.2021.790832)

# SUPPLEMENTARY FIGURES

## Supplementary Figure 1: Pie chart representing the allele frequency spectrum of 2,398,696 variants in the central Han Chinese population.

**
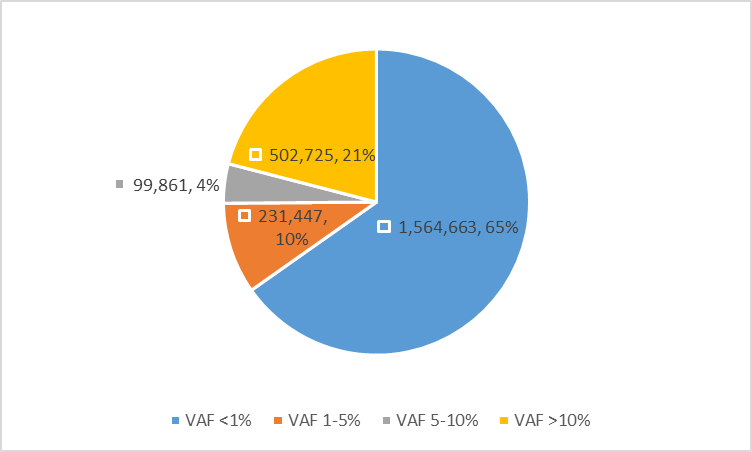
**

## Supplementary Figure 2：A Venn diagram representing the deleteriousness predictions for the nonsynonymous variants by three tools, SIFT, PolyPhen2 (PPH2), and Mutation Taster 2 (MT2).


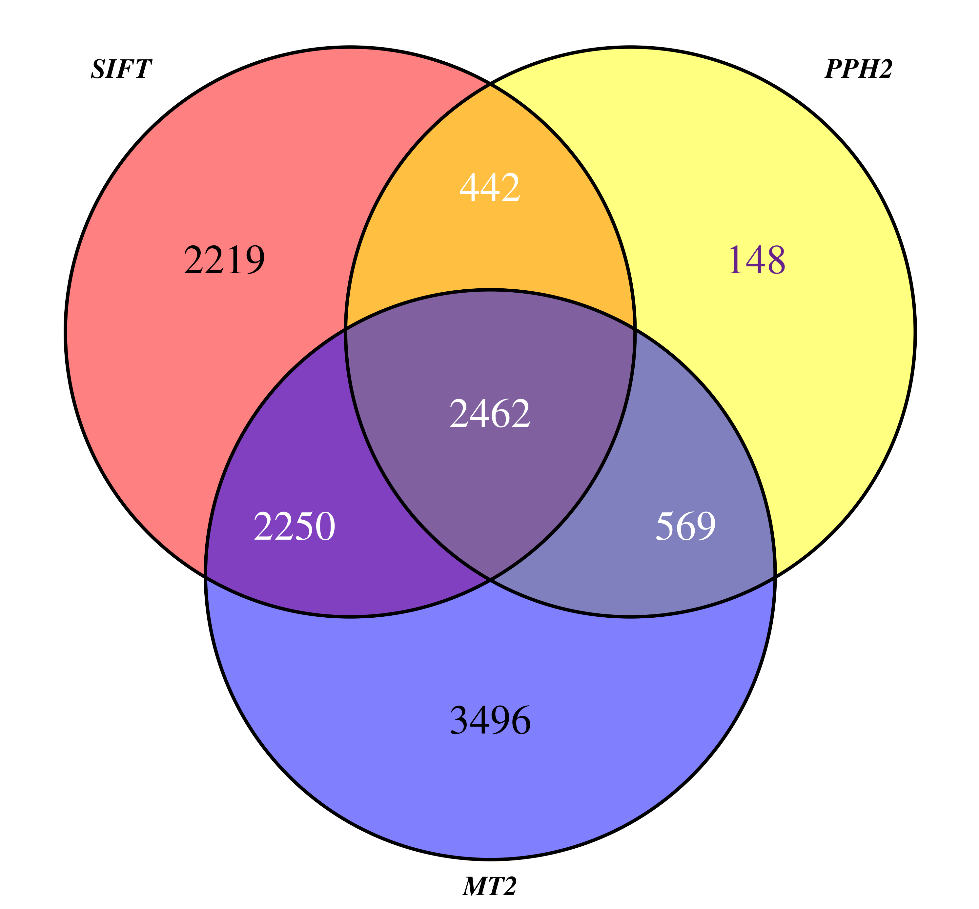


## Supplementary Figure 3: The allele frequency spectrum of potentially deleterious variants in the central Han Chinese population.


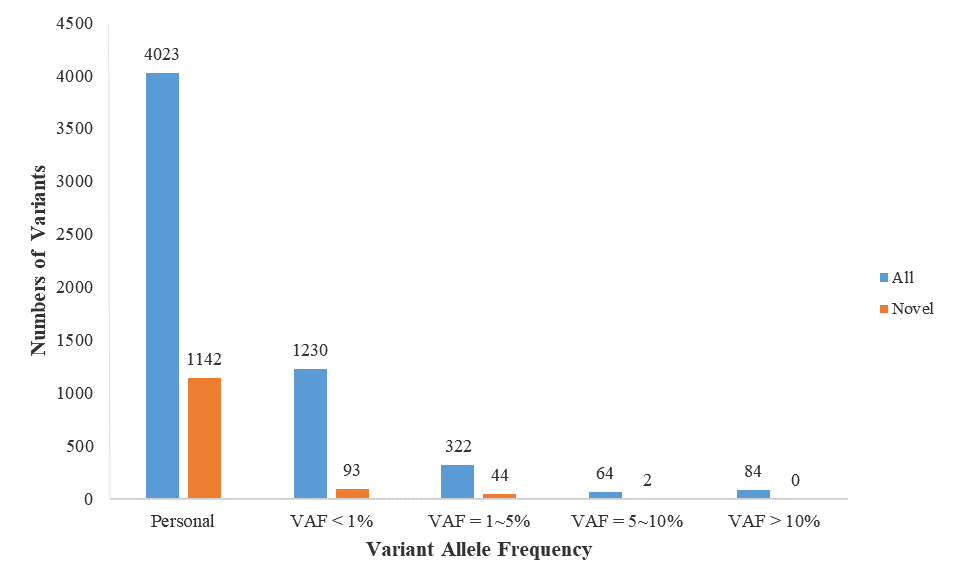


The “All” representing the allele frequency spectrum of the 5,724 potentially deleterious variants in the central Han Chinese population.

The “Novel” representing the allele frequency spectrum of the 1,282 deleterious “novel” variants in the central Han Chinese population.

## Supplementary Figure 4： Histogram representing the allele frequency spectrum of variants with PharmGKB clinical annotations in the central Han Chinese population.


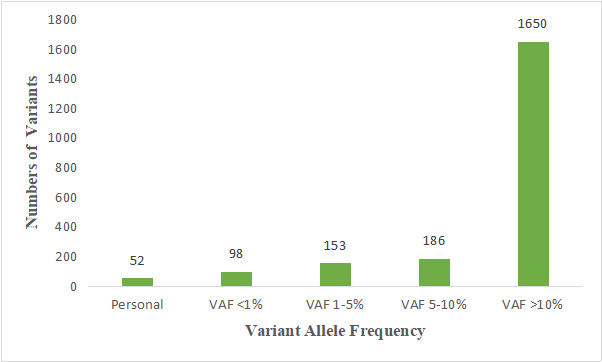

Supplement: Supplementary file 5 [file DataSheet1.docx]
